# The ICET-A Survey on Current Criteria Used by Clinicians for the Assessment of Central Adrenal Insufficiency in Thalassemia: Analysis of Results and Recommendations

**DOI:** 10.4084/MJHID.2016.034

**Published:** 2016-07-01

**Authors:** Vincenzo De Sanctis, Ashraf T. Soliman, Heba Elsedfy, Alice Albu, Soad Al Jaouni, Saif AL Yaarubi, Salvatore Anastasi, Duran Canatan, Massimo Di Maio, Salvatore Di Maio, Mohamed El Kholy, Mehran Karimi, Doaa Khater, Yurdanur Kilinc, Su Han Lum, Nicos Skordis, Praveen Sobti, Iva Stoeva, Ploutarchos Tzoulis, Yasser Wali, Christos Kattamis

**Affiliations:** 1 Pediatric and Adolescent Outpatient Clinic, Quisisana Hospital, Ferrara, Italy; 2 Department of Pediatrics, Division of Endocrinology, Hamad General Hospital Doha, Qatar and Department of Pediatrics, Division of Endocrinology, Alexandria University Children’s Hospital, Alexandria, Egypt; 3 Department of Pediatrics, Ain Shams University, Cairo, Egypt; 4 Endocrinology and Diabetes Department of Elias Hospital, Carol Davila University of Medicine and Pharmacy, Bucharest, Romania; 5 Head Division of Pediatric Hematology Oncology, Deputy Chair of Hematology & Head Section of Hematology Research Lab, King Fahd Medical Research Center Department of Hematology Faculty of Medicine, King Abdulaziz University Jeddah, Kingdom of Saudi Arabia; 6 Head of Pediatric Endocrine Unit, Department of Child Health, Sultan Qaboos University Hospital, Al-Khoud, Sultanate of Oman; 7 Thalassemia Unit, Maternal and Child Department, Garibaldi Hospital, Catania, Italy; 8 Director of Thalassemia Diagnosis Center of Mediterranean Blood Diseases Foundation Antalya, Turkey; 9 Department of Oncology, University of Turin, Mauriziano Hospital, Turin, Italy; 10 Emeritus Director in Pediatrics, Children’s Hospital “Santobono-Pausilipon”, Naples, Italy; 11 Hematology Research Center, Shiraz University of Medical Sciences, Shiraz, Iran; 12 Department of Pediatrics, Endocrinology Unit, Alexandria University Children’s Hospital, Egypt and Child Health Department, Sultan Qaboos University Hospital, Muscat, Oman; 13 Çukurova University, Medical Faculty, Department of Pediatric Hematology, Adana, Turkey; 14 Department of Paediatrics, University Malaya Medical Center, Malaysia; 15 Division of Pediatric and Adolescent Endocrinology, Paedi Center for Specialized Pediatrics, St. George’s University Medical School at the University of Nicosia, Cyprus; 16 Professor Pediatric Hemato-Oncology, Christian Medical College and Hospital, Ludhiana Punjab, India; 17 Paediatric Endocrinologist,”Screening and Functional Endocrine Diagnostics” SBALDB “Professor Ivan Mitev”, Medical University Sofia, Bulgaria; 18 Department of Endocrinology, Whittington Hospital, University College London, London, UK; 19 Pediatric Hematology Unit, Child Health Department, Sultan Qaboos University Hospital, Muscat, Oman and Department of Pediatrics, Alexandria University Children’s Hospital, Egypt; 20 First Department of Paediatrics, University of Athens, Athens, Greece

## Abstract

**Background:**

In March 2015, the International Network of Clinicians for Endocrinopathies in Thalassemia and Adolescent Medicine (ICET-A) implemented a two-step survey on central adrenal insufficiency (CAI) assessment in TM patients and after analysis of the collected data, recommendations for the assessment of hypothalamic-pituitary- adrenal (HPA) axis in clinical practice were defined.

**Methods:**

To ascertain the current practice for assessment of CAI in thalassemia, the Coordinator of ICET-A sent two questionnaires by email: i) The first to evaluate the current interpretation of basal serum cortisol level (*first step*) and ii) The second to assess the current usage of ACTH test and the variability in practice” (*second step*). Based on the surveys the core ICET-A group prepared the recommendations for the assessment of suspected CAI in thalassemia (*third step*).

**Results:**

A total of 19 thalassemologists/endocrinologists have participated in the first survey and 35 specialists participated in the second step questionnaire. The study demonstrated a considerable variability in almost all aspects of relevant current criteria used for the diagnosis of CAI. An ROC analysis using peak value > 20 μg/dl (> 550 nmol/L), after ACTH stimulation test, was performed with the aim of identifying the optimal basal serum cortisol cut-off. The optimal threshold that maximizes sensitivity plus specificity for morning basal cortisol against peak post-ACTH value >20 μg/dl (>550 nmol/L) was 10 μg/dl (275 nmol/L). Furthermore, the values associated with the highest negative predictive value (NPV) and highest, positive predictive value (PPV) were 4.20 (115 nmol/L) and 18.45 μg/dl (510 nmol/L), respectively.

Surprisingly, 20 specialists in thalassemia working in blood bank, thalassemia centres (day hospital), internal medicine, hematology and onco-hematology had poor knowledge and experience in testing for CAI and stopped filling the questionnaire after the second question. In contrast, 9 endocrinologists (8 pediatricians) and 6 hematologists working in collaboration with endocrinologists completed the questionnaire.

**Conclusions:**

While waiting for more extensive adequately powered and targeted studies, physicians should adopt an acceptable policy for accurate assessment of HPA in TM patients. Regular surveillance, early diagnosis, treatment and follow-up in a multi-disciplinary specialized setting are also recommended. The ICET-A recommendations are reported in order to facilitate for interested physicians the approach to a successful assessment of adrenal function in thalassemia.

## Introduction

Accurate assessment of the hypothalamic-pituitary- adrenal (HPA) axis is essential for the management of patients with potential or suspected pituitary or hypothalamic disease that is frequent in patients with thalassemia major (TM). The diagnosis of adrenal insufficiency (AI) is relatively simple when glucocorticoid secretion is profoundly depressed. However, AI can present a difficult diagnostic challenge, especially when adrenal insufficiency is partial. This is a particularly important issue as acute crises may occur during stress periods in undiagnosed patients.

Recently, several studies reported a significant prevalence of “biochemical” central adrenal insufficiency (CAI), ranging from 15% to 53.6 %,^1^–^5^ in children, adolescent and adults with TM. In one study the youngest patient reported with “biochemical” CAI was 9 years old.^4^ The age of patients varied from 12 and 20 years,^1^ 8 to 26 years,^2^ 10.2 ± 3.7 years (ranges are not available),^3^ 3 to 18 years^4^ and 18 to 50 years.^5^

The prevalence of CAI was higher in adult TM patients (32.1%; age range 18–50 years, median 30) (1–4). Extreme variability in the prevalence of CAI has been attributed to the variable duration of regular transfusion (p=0.016, 95% CI: −28.5/−3.24), iron overload status and the use of different tests for assessing adrenal function as well as different cut-off values for diagnosing AI among the various centres.^1^–^5^

The pathophysiological basis of “biochemical” AI in TM has not yet been well-defined. Chronic transfusions induce iron overload in several organs, including adrenal and pituitary glands.^1^–^5^ Therefore, it is possible that pituitary iron deposition might reduce ACTH secretion leading to CAI.^2^ Furthermore, the adrenal glands might also be directly affected by iron toxicity. In two studies, patients with TM had higher baseline adrenocorticotrophic hormone (ACTH) levels than do controls, suggesting primary impairment of adrenocortical function.^2^,^5^

There are two methods to differentiate between primary and secondary AI. First is done by measuring plasma ACTH concentration in the basal fasting AM blood sample. If it is higher than normal, the patient has primary AI, whereas if it is low, the diagnosis of secondary or tertiary AI should be considered. The second method assesses the serum cortisol values in response to exogenous corticotropin (ACTH) stimulation or insulin tolerance test (ITT). The agent most commonly used is synthetic ACTH [1–24] (cosyntropin), which has the full biologic potency of native ACTH [1–39]. The text is useful for the diagnosis of AI but not for the differential diagnosis between peripheral and central forms.^6^ Therefore, a prolonged corticotropin administration may become helpful in the differential diagnosis. Unfortunately, this diagnostic approach has not been validated in patients with TM.

In March 2015, the International Network of Clinicians for Endocrinopathies in Thalassemia and Adolescent Medicine (ICET-A) promoted a two- steps survey on the assessment CAI in TM patients among Endocrinologists and Hematologists working with thalassemia patients in different countries. After collecting and analysing the data, the ICET-A group prepared relevant clinical and practical recommendations for the assessment of HPA axis in these patients. The results of the ICET-A project are presented in this paper.

## Methods

To ascertain the current practice for assessment of CAI in thalassemia, the Coordinator (VDS) of ICET-A sent by email two questionnaires: i) “to evaluate the current interpretation of basal serum cortisol level” to 19 thalassemologists/endocrinologists members of ICET-A **(*first step*)** and ii) a copy of modified questionnaire survey used by Elder et al^7^ “to evaluate the current usage of ACTH test and the variability in practice” among other 20 additional specialists taking care of TM patients (***second step***). A total of 35 specialists participated in the second step questionnaire.

Based on the surveys the core ICET-A group prepared the recommendations for the assessment of suspected CAI (***third step***). The recommendations were based on published, peer-reviewed scientific evidence, expert opinion, and accumulated professional knowledge and experience. Recommendations from published guidelines were used when available and appropriate. The ICET-A Network also issued expert consensus opinions on topics for which limited or low level evidence is available in the literature. Since not all published references were based on randomised controlled trials, the recommendations have been scored according to the following criteria:

- **High confidence** indicates that further research is unlikely to change the confidence in the estimate of effect (●●●)
- **Moderate confidence** indicates that further research may change the confidence in the estimate of effect (●●○)
- **Low confidence** indicates that further research would likely have a significant impact on the confidence in the estimate of effect (●○○)
- **Insufficient** indicates that the evidence is unavailable or does not permit a conclusion (○○○)

## Statistical Analysis

A ROC analysis using peak value > 20 μg/dl (> 550 nmol/L) after ACTH stimulation test as the classification variable and basal value as the continuous predictor variable was performed using the data from the literature (2–4) and the personal experience, in 80 TM patients (aged 3–50 years) with the aim of identifying the optimal basal cut-off. The optimal cutoff was determined by the Youden Index, which is defined as Sensitivity plus Specificity-1. All analyses and calculations were done using R version 3.3.0, with the open-source package “pROC” (8).

## Results

### First step

Answered questionnaire was received from 15 out of 19 ICET-A members (78.9% response rate). Responders, who are collectively following 1895 TM patients, were asked to report their position on the lowest basal cortisol threshold used to diagnose CAI and the highest basal threshold excluding CAI. The results are summarized in table 1.

In the survey, the lowest basal cortisol threshold reported was ≤3 μg/dl (88 nmol/l) to exclude CAI and the highest threshold was ≤7 μg/dl (<190 nmol/l ) to diagnose CAI. Values greater than 20 μg/dl (550 nmol/l) were reported to predict best normal HPA axis.

**Figure 1:**
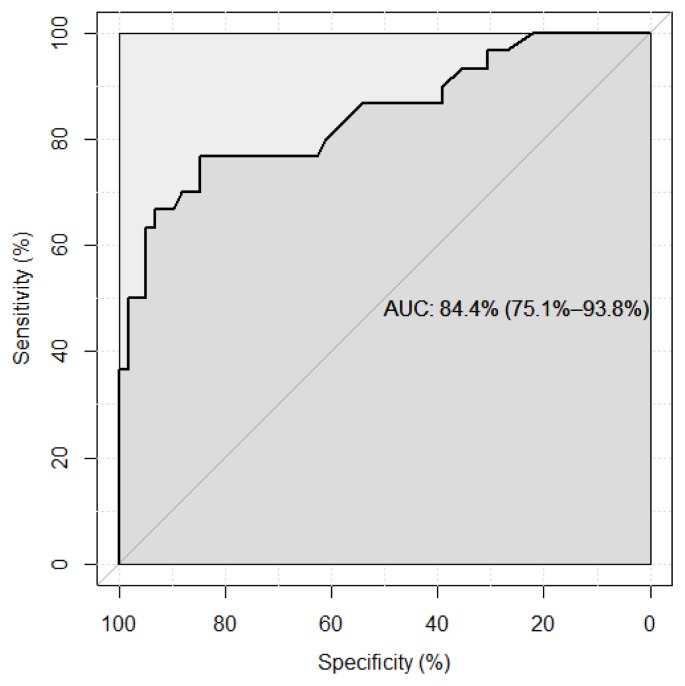
ROC curve using peak post-ACTH values of > 20 μg/dl ( >550 nmol/L.) as outcome.

The results of the ROC analysis are shown in table 2 and figure 1. Using the Youden index, the optimal threshold that maximizes sensitivity plus specificity for morning basal cortisol against peak post-ACTH value >20 μg/dl (>550 nmol/L) was 10 μg/dl (275 nmol/L). Three chemilumininescent assays (one Beckman Coulter and two Immulite 1000 kits) and one competitive enzyme-linked immunoassay were used for cortisol measurements (AccuBind kit). Furthermore, the values associated with the highest negative predictive value (NPV) and highest positive predictive value (PPV) were 4.20 (115 nmol/L) and 18.45 μg/dl (510 nmol/L), respectively.

### Second step

Thirty five centres following a total of 3433 TM patients shared the second questionnaire. Taking into consideration that several protocols have been used to assess the response to ACTH test, the aim of the survey was to collect 5 pieces of information regarding: a)How many patients are you regularly following in your hospital? b) Does your centre carry out Synacthen testing? Are you familiar with the indication and interpretation of Synacthen test, c) What dose of Synacthen does your Unit use? d) At what age you screen your patients by measuring serum cortisol? e) What cut off point you accept for diagnosing normal and abnormal adrenal function? f) For a normal result what do you require? (Table 3).

Surprisingly, 20 specialists working in blood banks, thalassemia centres (day hospital), internal medicine, hematology and onco-hematology had poor knowledge about the test and stopped filling the questionnaire after the second question. On the contrary 9 endocrinologists (8 were pediatricians) and 6 hematologists working in collaboration with endocrinologists completed the survey (Table 3). 9/15 (60 %) of responding centres employed a standard-dose (SDT) corticotropin stimulation test (Synacthen test: 250 μg intravenously) and 2/15 (26.6 %) a low dose (LDT) stimulation test (Synacthen test: 1 μg intravenously). There was a variation in the timing of cortisol sampling. 60 % collected blood samples at 0′, and at 30′ and 60′ minutes after ACTH injection. The maximum number of samples per test was 3. The diagnostic cut-off values used by different centres are reported in table 3. Peak cortisol level was used on its own as the diagnostic criteria in 60 % of centres and in association with cortisol rise from baseline in 26.6 % of centres. Peak cortisol value >18–20 μg/dl (200–550 nmol/l) was diagnostic for normal adrenal function in 10/15 centres (66.6%) while 5/15 centres (33.3%) required values > 20 μg/dl (550 nmol/l). Rise from baseline, defined as > 7 μg/dl (> 200 nmol/l) or 2–3 fold from baseline was required for diagnosing normal adrenocotical function by 40 % of the centres.

LDT were performed in 6/15 centres diluting one vial of 250 microgram ACTH into 250 mL sterile normal saline. 1 mL (=1 microgram ACTH) of the solution was then injected as an intravenous bolus.

## Discussion

Our survey demonstrated a considerable variability in the utilization of the current criteria used for the diagnosis of AI. These included serum basal cortisol level, ACTH dose, the timing of cortisol sampling and cut-offs for AI. In our study, the optimal threshold that maximizes sensitivity plus specificity for morning basal cortisol against peak post-ACTH value >20 μg/dl (>550 nmol/L) was 10 μg/dl (275 nmol/L).

Furthermore, the values associated with highest negative predictive value (NPV) and highest positive predictive value (PPV) were 4.20 (115 nmol/L) and 18.45 μg/dl (510 nmol/L), respectively.

The lower cut-off is in line with the published data while the upper cut-off is markedly lower than the one reported in the meta-analysis.^9^ In fact, in a meta-analysis of 12 studies on adults (635 subjects without thalassemia), a basal cortisol less than 5 μg/dl (< 138 nmol/l) strongly predicted CAI, while values greater than 13 μg/dl (365 nmol/l) a normal HPA axis.^9^

The lack of uniformity in cut-off levels could in part be attributed to differences in study populations, the variability of dynamic tests, different serum cortisol assays used, the cut-off of peak serum cortisol that was deemed indicative of a normal HPA axis response, and the clinical context in which the studies were done. Therefore, additional studies are required to further elucidate these differences.

Dynamic testing is performed to establish the diagnosis in patients with equivocal cortisol levels in whom hypoadrenalism is suspected. Several protocols have been used to assess the response to exogenous corticotrophin (ACTH). The agent used is synthetic ACTH [1–24] (cosyntropin), which has the full biologic potency of native ACTH [1–39]. There is controversy whether the low-dose test (LDT) is superior to the high-dose ACTH stimulation test (HDT).

The existing controversies in the literature about the use of different Synachten stimulation tests in the assessments of the HPA axis are thought to be related to the use of inappropriate cut-off values.^1^–^5^,^10^–^14^ Conventionally, adrenal insufficiency is likely if serum cortisol level is less than 18–20 μg/dL (500–550 nmol/L) at 30–60 minutes after administration of ACTH and or an increments of less than 7 μg/dl (200 nmol/L) above basal cortisol, a criterion described by Crowley et al for LDT (10). Olkers et al (11) suggested the importance of using different cut-off points for HDT and LDT. A raised cut-off of 22 μg/dl (600 nmo/l) could result in higher sensitivity for the diagnosis.

Mayenknecht et al.^12^ established normal ranges for cortisol responses in the LDT (0.5 mg/m^2^ tetracosactin injection) and HDT in 35 endocrinologically normal healthy subjects. Mean responses minus 2 standard deviations were used as the cut-off point. The result for the LDT at 30 min after injection was 20 μg/dL (550 nmol/L); for the HDT at 30 min after injection: 22 μg/dL (600 nmol/L) and at 60 min: 26 μg/dL (715 nmol/L). The authors concluded that it was crucial to use different cut-off points in the HDT and LDT tests.

It is possible that TM patients with mild/partial or recent-onset pituitary ACTH or hypothalamic corticotropin-releasing hormone (CRH) deficiency may have a normal response to 250 μg of Synacthen because the adrenal glands have not undergone significant atrophy and still responds to very high concentrations of ACTH (13,14). This is an especially important issue because acute crisis may occur during stress periods in undiagnosed patients

A meta-analysis including 30 studies (1209 adults and 228 children) compared the results of high- and low-dose ACTH stimulation tests using different peak serum cortisol cut-offs. The analysis showed that both tests had similar diagnostic accuracy in adults and children. In general, both tests had low sensitivity and high specificity resulting in reasonable likelihood ratios for a positive test, but a relatively suboptimal likelihood ratio for a negative test.^15^ Another survey, published in 2016, supports that there is no clear evidence to indicate that one test is superior to another.^16^ This report conflicts with earlier studies with a small number of patients, suggesting that the low-dose test was more sensitive.^17^–^21^

In patients with TM, an AI was demonstrated in 30 of 56 patients (53.6%) after an LDT. To assess more precisely the adrenal function, the insulin tolerance test (ITT) was performed in 26 of 30 TM patients (86.7%) who had peak total cortisol less than 16 μg/dl (440 nmol/l), after ACTH test. The remaining four patients declined the testing. The time interval between the 1 μg ACTH test and ITT was approximately 4–5 wk. Five of 26 patients (19.2%) had peak total cortisol after an ITT of 20 μg/dl or greater. Therefore, about one fifth of patients who failed the 1 μg ACTH test had normal peak total cortisol levels after an ITT. Thus, by using an ITT, the estimated frequency of adrenal insufficiency in the entire patient group was reduced by approximately 20%.^3^

Soliman et al.^2^ using the apparently more “physiologic” LDT and a normal peak total cortisol cut-off level of 20 μg/dl (550 nmol/L) and increment >7 μd/dl (>200 nmol/l), diagnosed a prevalence of CAI in 8 out of 23 (34.7%) in TM patients (6 adolescents and 2 children). Using the HDT and the same cut-off levels diagnosed AI in 8.7% (2/23) of these adolescents. Therefore, about 75% of patients who failed the LDT had normal peak total cortisol levels after the HDT. Similar results were reported by Pang et al. in 6/8 TM patients.^4^

In conclusion, further studies and more normative data are urgently needed because neither over diagnosis nor under diagnosis of HPA insufficiency should be acceptable in patients who potentially may be treated with steroids unnecessarily or who may have impaired cortisol in times of stress and are in need of steroids.^2^,^22^

Twenty specialists working in blood bank, thalassemia centres (day hospital), internal medicine, hematology, and onco-hematology had poor knowledge of the test and stopped to fill the questionnaire after the second question. On the contrary, 9 endocrinologists and 6 hematologists working in collaboration with endocrinologists completed the survey questionnaire.

Therefore, a collaborative working arrangement between professionals is needed to meet all the required comprehensive care to patients. We believe that one of the near future ICET-A tasks is to set up a collaborative enterprise to identify and address the underlying factors that lead to barrier inter-professional team work and thereby to facilitate inter-professional collaboration.

The lack of treatment guidelines and published research often leave hematologists and internists with hesitant to approach TM patients presenting uncommon endocrine complications. Therefore, as a ***third step,*** we thought worth to prepare clinical practice recommendations for all those taking care of TM patients on current criteria for the assessment of CAI (Table 4). The recommendations provide helpful information on laboratory parameters and their interpretation, as well as adrenal hormone replacement dosages and management strategies. The guidelines emphasize that clinicians need to suspect AI earlier in TM patients with risk factors, such as advanced age, severe iron overload and/or poor compliance to therapy, and with multiple endocrine complications.

If corticotropin testing is not feasible, a combination of a morning plasma ACTH and cortisol levels (less than 4.2 μg/dL = 115 nmo/L) can be used as an initial screening; based on the results, a confirmatory testing with corticotropin stimulation is strongly recommended. Because tests are not perfect, there is still an important role for clinical judgment, especially regarding the use of glucocorticoid supplementation during extreme stress, such as surgery.^2^,^22^

In summary, our survey provides a better understanding of current physician clinical practices and beliefs in the assessment of the hypothalamic-pituitary-adrenal axis in TM patients. While waiting for more extensive, adequately powered and targeted studies, physicians should adopt an applicable, common sense policy for accurate assessment of HPA in TM patients. Regular surveillance, early diagnosis, treatment and follow-up in a multi-disciplinary specialized setting are also recommended.

## Figures and Tables

**Table 1:** Criteria used for the interpretation of basal cortisol levels (expressed in μg/dl) in 15 Centres taking care of 1895 thalassemia major patients.

Countries	High probability of AI	Low probability of AI	Methods
Bulgaria	< 5 μg/dl (^*^)	> 20 μg/dl (^*^)	Siemens-Immulite-2000 Chemiluminescent assay
Cyprus	< 5 μg/dl	> 10 μg/dl	Siemens-Immulite-2000 Chemiluminescent assay
Egypt	< 3 μg/dl	> 15 μg/dl	AccuBind Enzyme Immunoassay
India	< 3 μg/dl	> 18 μg/dl	Roche-Elecsys Chemiluminescence assay
Iran	< 5 μg/dl	> 15 μg/dl	Radim Diagnostic Chemiluminescence assay
Italy	< 4 μg/dl	> 14 μg/dl	Immulite-1000 Chemiluminescence assay
Italy	< 3 μg/dl	> 16 μg/dl	Immulite-1000 Chemiluminescence assay
Italy	< 7 μg/dl	> 14 μg/dl	Abbott Chemiluminescence assay
Malaysia	< 3.6 μg/dl	> 20 μg/dl	Advia-Centaur Chemiluminescence assay
Oman	< 7 μg/dl	> 10 μg/dl	Beckman Coulter Chemiluminescence assay
Qatar	< 7 μg/dl	> 10 μg/dl	Immulite-1000 Chemiluminescence assay
Romania	< 5 μg/dl	> 15 μg/dl	Immulite-2000 Chemiluminescence assay
Saudi Arabia	< 7 μg/dl	> 18 μg/dl	Siemens-Immulite-2000 Chemiluminescent assay
Turkey	< 7 μg/dl	> 10 μg/dl	Beckman Coulter Chemiluminescence assay
UK	< 3.6 μg/dl	> 16 μg/dl	Abbott Chemiluminescence assay

(*) multiply by 27.57 to convert μg/dl to nmol/L

**Table 2:** ROC analysis ruling out CAI showing sensitivity, specificity, positive predictive value (PPV) and negative predictive value (NPV) for different basal serum cortisol levels.

**Threshold (baseline cortisol value,μg/dl)**	**4.20**	4.75	5.85	6.40	7.90	8.05	9.95	10.7	12.8	14.7	15.7	**18.45**
**Sensitivity**	**100**	96.67	93.33	90.00	86.67	80.00	76.67	70.00	66.67	63.33	50.00	**36.67**
**Specificity**	**22.03**	30.51	35.59	38.98	54.23	61.01	84.75	88.14	93.22	94.92	98.31	**100.00**
**PPV**	**39.47**	41.43	42.42	42.86	49.06	51.06	71.87	75.00	83.33	86.36	93.75	**100.00**
**NPV**	**100**	94.74	91.30	88.46	88.89	85.71	87.72	85.25	84.62	83.58	79.45	**75.64**

**Legend**: PPV: positive predictive value; NPV: negative predictive value

**Table 3:** Results of Synacthen test questionnaire completed by 15 Centres.

Survey questionnaire	Endocrinologists

1. How many patients are you regularly following?	1895 (50–500)
Total number (Minimum-Maximum )	

2. What dose of Synacthen does your unit use?	
Low dose (1 μg)	2/15
Standard dose (250 μg)	9/15
Both	4/15
A different dose	**-**

3. At what time do you take your cortisol samples?	
0 min	15/15
10 min	-
30 min	1/15
60 min	3/15
30 and 60 min	9/15
20 and 60 min	1/15
20,30 and 60 min	1/15

4. What cut off for normal do you use?	
a. Peak cortisol :	5/15
> 18 μg/dl (^*^)	5/15
> 20 μg/dl	4/15
> 21 μg/dl	1/15
other (22 μg/dl )	
b. Rise from baseline:	
>7 μg/dl	5/15
Other (2–3 fold from baseline)	1/15

5. For a normal result do you require :	
a. Only peak cortisol	9/15
b. Only peak rise from baseline	-
c. Both peak and rise from baseline	4/15
d. Either peak or baseline but not both	2/15

(*) multiply by 27.57 to convert μg/dl in nmol/L

**Table 4:** Practical recommendations for the assessment of suspected adrenal insufficiency (AI) in thalassemia

**Who should be investigated?** TM patients with high risk for ACTH deficiency *e.g*. patients in advanced age, subjects with severe iron overload or poor compliance to therapy and patients with multiple endocrine complications.^1^–^5^ (●●○) **How is adrenal insufficiency diagnosed?** The first step consists of a single early morning plasma ACTH and serum cortisol values (before 9 AM). The diagnosis of primary AI is established with a low serum cortisol combined with an elevated plasma ACTH (> 2-fold the upper limit of the reference range). If ACTH is also low, the diagnosis of secondary or tertiary AI is considered.^13^ (●●○) The basal serum cortisol values are dependent on the assay used and should be validated against a local reference population. (●●○) **Basal serum cortisol cut-off level:** A serum cortisol concentration greater than 10 μg/dL (276 nmol/L) makes it unlikely that the patient has clinically meaningful HPA insufficiency. In addition, a value below 4.2 μg/dL (115 nmol/L) makes AI very likely ( see Table 2). Serum basal cortisol level within the normal range does not exclude the diagnosis of mild/partial adrenal insufficiency.^22^–^26^ (●●○) **What other tests should be performed after suspected diagnosis of AI ?** Dynamic function tests should be performed when there is doubt about the status of hypothalamic-pituitary-adrenal (HPA) function. The test can be carried out as an outpatient at any time. The HPA axis response to insulin tolerance test (ITT) is still considered the gold standard in the evaluation of suspected AI (●●●). However, this test is potentially dangerous necessitating therefore hospitalization and continuous supervision and their utilization are limited by several contra-indications.^27^ (●●○) Once AI has been established, CRH stimulation test is another endocrine function test that directly stimulates ACTH secretion from the pituitary gland, and subsequently cortisol secretion. A normal response does not rule out partial tertiary adrenal insufficiency, but an abnormal response can help to localize the defect.^28^ (●○○) Although glucagon stimulation test (GST) represents an alternative to the ITT as a screening test for central AI, further larger studies are required to assess the cut-off cortisol level accurately for diagnosing an AI.^29^ (●○○) **Standard high-dose versus low- dose ACTH stimulation test:** There is still controversy regarding whether the LDT is superior to the SDT and whether SDT predicts the ability of a patient to respond adequately to stress, such as major surgery. Therefore, SDT is not recommended either in patients with partial ACTH deficiency or when the time of start of the deficiency is unknown.^13^,^14^ (●●○) The low-dose ACTH test (LDT) is performed by measuring serum cortisol immediately before and 20–30 minutes after IV injection of cosyntropin (synthetic ACTH). There is no commercially available preparation of “low-dose” cosyntropin. Therefore, low-dose (1 μg) corticotropin test requires dilution of the supplied corticotropin to the required dose, which can introduce dosing errors and sources of contamination into the diagnostic procedure. (●●○) A standard high-dose test (SDT) is performed by measuring serum cortisol before, 30 and 60 minutes after intravenous (IV) injection of 250 μg of cosyntropin. (●●○) **Interpretation of serum cortisol response:** A minimum serum cortisol concentration > 18–20 μg/dL (550 nmol/L) before or after corticotropin (ACTH) injection virtually exclude AI. Peak serum cortisol levels less than 18 μg/dL has 97.5% sensitivity and 95% specificity for diagnosis of AI.^30^ (●●○) **What other tests might a health care provider perform after diagnosis of CAI?** LDT and SDT are screening test, and abnormal responses need to be followed up with further tests (investigation of anterior pituitary function and MRI of the hypothalamic-pituitary region for the assessment of iron overload). (●●○) **Severely ill patients.** Reliable assessment of hypothalamic-pituitary-adrenal axis reserve is difficult in severely ill patients because cortisol-binding globulin (CBG) levels fall substantially during the acute phase response. 80% of total cortisol is bound to CBG and variation in CBG significantly affects total cortisol levels, which should be interpreted with caution.^13^ (●●○) **What precautions should be considered when performing a stimulation test**? Patients on oral contraceptives or on hormone replacement therapy, which increase cortisol-binding globulin (CBG) levels, should stop this 6 weeks prior to the test.(●●○); Pregnant woman. The diagnosis of adrenal insufficiency in pregnancy remains challenging. Baseline serum total cortisol concentration in late gestation can be misleading, especially if the criteria used for diagnosis are based on norms determined in non-pregnant women (who have markedly lower CBG levels).^13^ (●●○) **Contraindication to ACTH stimulation test:** ACTH test is contraindicated in subjects with previous untoward reaction to Synacthen. (●●○)

## References

[1] Elsedfy HH, El Kholy M, Tarif R, Hamed A, Elalfy M (2011). Adrenal function in thalassemia major adolescents. Pediatr Endocrinol Rev.

[2] Soliman AT, Yassin M, Majuid NM, Sabt A, Abdulrahman MO, De Sanctis V (2013). Cortisol response to low dose versus standard dose (back-to-back) adrenocorticotrophic stimulation tests in children and young adults with thalassemia major. Indian J Endocrinol Metab.

[3] Poomthavorn P, Isaradisaikul B, Chuansumrit A, Khlairit P, Sriphrapradang A, Mahachoklertwattana P (2010). High prevalence of “biochemical” adrenal insufficiency in thalassemics: Is it a matter of different testings or decreased cortisol binding globulin?. J Clin Endocrinol Metab.

[4] Pang GSW, Lee CY, Ling ASC, Leung WC, Yau HC (2015). Adrenal Insufficiency in Paediatric Transfusion Dependent Thalassaemia Major in Hong Kong: A Pilot Study. HK J Paediatr (New Series).

[5] Scacchi M, Danesi L, Cattaneo A, Valassi E, Pecori Giraldi F, Radaelli P, Ambrogio A, D’Angelo E, Mirra N, Zanaboni L, Cappellini MD, Cavagnini F (2010). The pituitary-adrenal axis in adult thalassaemic patients. Eur J Endocrinol.

[6] Charmandari E, Nicolaides NC, Chrousos GP (2014). Adrenal insufficiency. Lancet.

[7] Elder CJ, Sachdev P, Wright NP (2012). The short Synacthen test: a questionnaire survey of current usage. Arch Dis Child.

[8] Robin X, Turck N, Hainard A, Tiberti N, Lisacek F, Sanchez JC, Müller M (2011). pROC: an open-source package for R and S+ to analyze and compare ROC curves. BMC Bioinformatics.

[9] Kazlauskaite R, Evans AT, Villabona CV, Abdu TA, Ambrosi B, Atkinson AB, Choi CH, Clayton RN, Courtney CH, Gonc EN, Maghnie M, Rose SR, Soule SG, Tordjman K, Consortium for Evaluation of Corticotropin Test in Hypothalamic-Pituitary Adrenal Insufficiency (2008). Corticotropin tests for Hypothalamic-Pituitary-Adrenal Insuffciency: A Metaanalysis. J Clin Endocrinol Metab.

[10] Crowley S, Hindmarsh PC, Holownia P, Honour JW, Brook CGD (1991). The use of low dose of ACTH in the investigation of adrenal function in man. J Endocrinol.

[11] Oelkers W (1998). The role of high- and low-dose corticotropin tests in the diagnosis of secondary adrenal insufficiency. Eur J Endocrinol.

[12] Mayenknecht J, Diederich S, Bähr V, Plöckinger U, Oelkers W (1998). Comparison of low and high dose corticotropin stimulation tests in patients with pituitary disease. J Clin Endocrinol Metab.

[13] Halperin Rabinovich I, Obiols Alfonso G, Soto Moreno A, Torres Vela E, Tortosa Henzi F, Català Bauset M, Gilsanz Peral A, Girbés Borràs J, Moreno Esteban B, Picó Alfonso A, Del Pozo, Picó C, Zugasti Murillo A, Lucas Morante T, Páramo Fernández C, Varela da Sousa C, Villabona Artero C (2008). Clinical practice guideline for hypotalamic-pituitary disturbances in pregnancy and the postpartum period. Endocrinol Nutr.

[14] Dickstein G, Arad E, Schechner C (1997). Low dose ACTH stimulation test. Endocrinologist.

[15] Ospina NS, Al Nofal A, Bancos I, Javed A, Benkhadra K, Kapoor E, Lteif AN, Natt N, Murad MH (2016). ACTH Stimulation Tests for the Diagnosis of Adrenal Insufficiency: Systematic Review and Meta-Analysis. Clin Endocrinol Metab.

[16] Ng SM, Agwu JC, Dwan K (2016). A systematic review and meta-analysis of Synacthen tests for assessing hypothalamic-pituitary-adrenal insufficiency in children. Arch Dis Child.

[17] Tordjman K, Jaffe A, Grazas N, Apter C, Stern N (1995). The role of the low dose (1 microgram) adrenocorticotropin test in the evaluation of patients with pituitary diseases. J Clin Endocrinol Metab.

[18] Dickstein G (1998). Commentary to the article: comparison of low and high dose corticotropin stimulation tests in patients with pituitary disease. J Clin Endocrinol Metab.

[19] Zarkovic M, Ciric J, Stojanovic M, Penezic Z, Trbojevic B, Drezgic M, Nesovic M (1999). Optimizing the diagnostic criteria for standard (250-microg) and low dose (1-microg) adrenocorticotropin tests in the assessment of adrenal function. J Clin Endocrinol Metab.

[20] Abdu TA, Elhadd TA, Neary R, Clayton RN (1999). Comparison of the low dose short synacthen test (1 microg), the conventional dose short synacthen test (250 microg), and the insulin tolerance test for assessment of the hypothalamo-pituitary-adrenal axis in patients with pituitary disease. J Clin Endocrinol Metab.

[21] Mayenknecht J, Diederich S, Bähr V, Plöckinger U, Oelkers W (1998). Comparison of low and high dose corticotropin stimulation tests in patients with pituitary disease. J Clin Endocrinol Metab.

[22] Banani SA, Omrani GH (2000). Cortisol and adrenocorticotropic hormone response to surgical stress (splenectomy) in thalassemic patients. Pediatr Surg Int.

[23] Hägg E, Asplund K, Lithner F (1987). Value of basal plasma cortisol assays in the assessment of pituitary-adrenal insufficiency. Clin Endocrinol (Oxf).

[24] Yip CE, Stewart SA, Imran F, Clarke DB, Mokashi A, Kaiser SM, Imran SA (2013). The role of morning basal serum cortisol in assessment of hypothalamic pituitary-adrenal axis. Clin Invest Med.

[25] Schmidt IL, Lahner H, Mann K, Petersenn S (2003). Diagnosis of adrenal insufficiency: Evaluation of the corticotropinreleasing hormone test and Basal serum cortisol in comparison to the insulin tolerance test in patients with hypothalamic-pituitary-adrenal disease. J Clin Endocrinol Metab.

[26] Le Roux CW, Meeran K, Alaghband-Zadeh J (2002). Is a 0900-h serum cortisol useful prior to a short synacthen test in outpatient assessment?. Ann Clin Biochem.

[27] Reimondo G, Bovio S, Allasino B, Terzolo A (2008). Secondary hypoadrenalism. Pituitary.

[28] Huang KE, Mittelman SD, Coates TD, Geffner ME, Wood JC (2015). A significant proportion of thalassemia major patients have adrenal insufficiency detectable on provocative testing. J Pediatr Hematol Oncol.

[29] De Sanctis V, Elsedfy H, Soliman AT, Elhakim IZ, Soliman NA, Karimi M, Elalaily R (2016). The Diagnostic Approach to Central Adrenocortical Insufficiency (CAI) in Thalassemia. Mediterr J Hematol Infect Dis.

[30] Dorin R, Qualls CR, Crapo LM (2003). Diagnosis of adrenal insufficiency. Ann Intern Med.

